# Atomic scale investigation of silicon nanowires and nanoclusters

**DOI:** 10.1186/1556-276X-6-271

**Published:** 2011-03-30

**Authors:** Manuel Roussel, Wanghua Chen, Etienne Talbot, Rodrigue Lardé, Emmanuel Cadel, Fabrice Gourbilleau, Bruno Grandidier, Didier Stiévenard, Philippe Pareige

**Affiliations:** 1 Groupe de Physique des Matériaux, Université et INSA de Rouen, UMR CNRS 6634 - Av. de l'université, BP 12, 76801 Saint Etienne du Rouvray, France; 2 Centre de Recherche sur les Ions, les Matériaux et la Photonique, UMR CNRS 6252/CEA/ENSICAEN/UCBN, 6 Bd. Maréchal Juin, 14050 Caen Cedex 4, France; 3 Institut d'Electronique, de Microélectronique et de Nanotechnologie, UMR CNRS 8520, Département ISEN, 41 bd Vauban, 59046 Lille Cedex, France

## Abstract

In this study, we have performed nanoscale characterization of Si-clusters and Si-nanowires with a laser-assisted tomographic atom probe. Intrinsic and *p*-type silicon nanowires (SiNWs) are elaborated by chemical vapor deposition method using gold as catalyst, silane as silicon precursor, and diborane as dopant reactant. The concentration and distribution of impurity (gold) and dopant (boron) in SiNW are investigated and discussed. Silicon nanoclusters are produced by thermal annealing of silicon-rich silicon oxide and silica multilayers. In this process, atom probe tomography (APT) provides accurate information on the silicon nanoparticles and the chemistry of the nanolayers.

## Introduction

Low-dimensional nano-structured materials, such as carbon nanotubes [1], silicon nanowires (SiNWs) [2], and silicon nanoclusters (SiNCs) [3], have attracted much interest in recent years because of their special properties (electrical, optical, mechanical, etc.) compared to bulk materials. The morphology and number density of nanoclusters as well as the dopant or impurity concentration and their spatial distributions in nanowires can greatly affect their properties. Thus, a key issue that remains is to analyze and characterize nano-structured materials at the atomic scale.

In this study, we have used the laser-assisted wide angle Tomographic Atom Probe to characterize SiNWs and SiNCs, respectively. The atom probe tomography (APT) involves the use of a three-dimensional (3D) high-resolution analytic microscope that can map the spatial distribution of atoms in materials at the atomic scale. The principle of the APT is based on the field evaporation of atoms. A conventional APT relies on the basic principle of the field evaporation of atoms from the surface of a specimen under high-voltage (HV) pulses [4,5]. The chemical nature of each evaporated ion is determined by the time of flight mass spectrometry using a position-sensitive detector (PSD). The set of information (position and chemical nature) allows for the 3 D reconstruction of the ionized volume. The specimen must be prepared in the form of a sharp needle with a diameter smaller than 100 nm to generate a sufficient electric field at the apex to favor the ionization and evaporation of atoms during HV pulses. In the case of poor conductive materials such as semiconductor NWs, HV pulses are replaced by femtosecond laser pulses to evaporate the semiconducting materials. This is the so called laser-assisted APT. The laser pulse frequency triggers the evaporation of atoms from the surface of the specimen toward the PSD. This information is used for the 3 D reconstruction of the material at the atomic scale as previously mentioned. A detailed description of the laser-assisted APT can be found in [6-8].

Several methods can be utilized for the preparation of a very sharp nano-tip with a diameter smaller than 100 nm. Considering the preparation of SiNWs, a two-step process can be proposed: (i) choosing under optical microscopy a suitable SiNW (diameter, direction, length, etc.) from the Si-growing substrate covered with SiNWs using a pre-prepared W tip; and (ii) welding the SiNW and the W tip in a dual beam system [scanning electron microscope (SEM)-gas injection system and focused ion beam (FIB)] using Pt as solder. As far as the preparation of SiNCs for APT investigation is considered, a conventional lift-out sample preparation can be proposed. The SiNC-based materials are sputtered on a Si substrate and formed by annealing treatments. Using a classical lift-out method, a multilayer (ML) chunk can be mounted on a pre-prepared steel needle [9]. The APT tip is then prepared by annular milling using the FIB [10].

## Investigation of SiNWs

SiNWs are 1 D nano-structures which can be applied in various domains, such as solar cells [11] and biosensor [12]. Either of the two types of approaches can be used to elaborate SiNWs: "top-down" or "bottom-up". In this study, we have studied the SiNWs synthesized by bottom-up approach. Several mechanisms are proposed based on this approach. Among them, the vapor liquid solid mechanism, proposed by Wagner and Ellis in 1964 [13], is the most widely used. According to this mechanism, the growth process of SiNWs involves three elements: reactant, catalyst, and substrate, and three processing steps: supply of Si atoms from reactant, incorporation of Si atoms into the catalyst droplet, and crystallization of Si atoms at the catalyst/substrate interface. Considering step 1, two methods can be utilized: chemical vapor deposition (CVD) and molecular beam epitaxy. Here, CVD was used because it allows for a better control of the growth condition and doping incorporation (addition of diborane silane). However, the true concentration of doping, its spatial distribution, and the presence or absence of impurities (catalyst atoms...) remain as experimental bottlenecks. In this study, Au was used as catalyst to grow SiNWs. Au is chosen because of its low eutectic temperature when mixed with Si and its easy preparation in the form of a homogeneous distribution of droplets at the surface of the silicon substrate. However, owing to its atomic diffusion [14], Au atoms can create intense deep traps in SiNW and, therefore, decrease the electrical transport [15]. Thus, the investigation of the presence and the location of Au atoms in SiNW remain an important issue. The laser-assisted APT has been widely used to investigate this aspect.

### Intrinsic SiNWs

**Figure 1:**
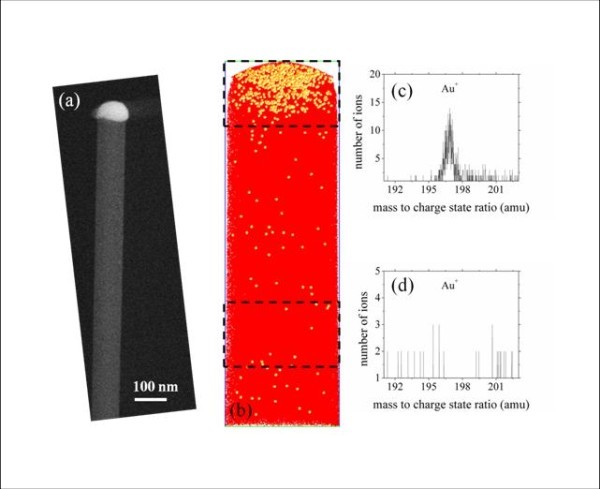
**Laser-assisted APT investigation of gold atoms in SiNWs**. **(a) **SEM image of an individual SiNW. The gold droplet is clearly detected at the top end of SiNW. **(b) **A slice of the 3 D reconstruction volume of one part of SiNW characterized by the laser-assisted APT. Each dot represents one atom in the reconstruction volume. Si and Au atoms are represented by red and yellow dots, respectively. The slice surface is 32 × 130 nm^2^. **(c,d) **Mass spectra of two regions in SiNW: top end and interior (the dashed box in **b**) of SiNW. The Au^+ ^peak can be clearly detected in mass spectrum at the top end region of SiNW.

The detection of Au atoms in SiNWs has been first performed in intrinsic SiNW. The catalyst, gold droplet, is prepared using evaporation of gold atoms on a Si substrate. The growth temperature and time are 500°C and 30 min. respectively. Silane is diluted with hydrogen in the ratio of 50:49. The flow rate is 50 standard cubic centimeter per minute (sccm), and the silane partial pressure is kept at 0.4 mbar during SiNW growth. The diameter of Au droplets is controlled to be less than 100 nm to allow the morphology of SiNWs being adapted to APT investigation without any post treatment such as FIB milling. A single SiNW is presented in Figure 1a. The Au droplet can be seen on the top end of the SiNW. Figure 1b shows a slice of the 3 D reconstruction of a part of the SiNW. The surface of the slice is 32 × 130 nm^2^. Each dot in the reconstruction volume is one atom. Red and yellow dots represent Si and Au atoms, respectively. As indicated in Figure 1b, Au atoms are mainly detected in the droplet located on the top end of SiNW, and very few Au atoms are detected within the SiNW. To be more precise, the signal (mass pick) associated to gold atom in the SiNW does not emerge from the background noise of the experimental mass spectrum. In other words, if gold is present in the SiNW, the atomic level is below 5 ± 0.5 × 10^17 ^at./cm^3^. The mass-to-charge ratio spectra of SiNW investigated by APT is presented in Figure 1c, d for the top end and the core region respectively.

### *P*-type SiNWs

**Figure 2:**
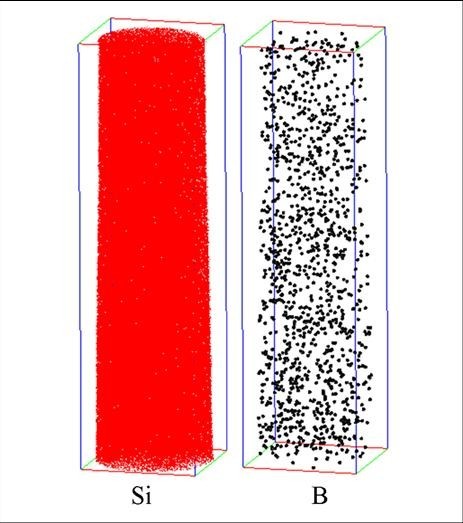
**Three-dimensional reconstruction of one part of *p*-type SiNW characterized by laser-assisted APT**. The reconstruction volume is 16 × 16 × 58 nm^3^. The red and black dots represent Si and B atoms, respectively. From the reconstruction, it can be seen that, B atoms are homogeneously distributed in the SiNW core with a relatively high concentration: 1.4 ± 0.1 × 10^20 ^B/cm^3^.

The *p*-doped SiNW is also characterized by laser-assisted APT. We chose diborane as the *p*-type dopant reactant, and Au and silane as the catalyst and the Si precursor, respectively. The growth temperature and the time of SiNW are fixed as 500°C and 30 min, respectively. Silane is diluted with hydrogen in a ratio of 50:49, and the flow rate of diborane is 1 sccm. Figure 2 represents the 3 D reconstruction of a *p*-type SiNW investigated by APT. The reconstruction volume is 16 × 16 × 58 nm^3^. The red and black dots represent Si and B atoms, respectively. The B concentration is 1.4 ± 0.1 × 10^20 ^B/cm^3^. It can be seen from Figure 2 that B atoms are homogeneously distributed in the core of the SiNW with a relatively high doping concentration.

## Characterization of SiNCs embedded in silica

SiNCs have been extensively studied since the discovery of photoluminescence (PL) of porous silicon [16]. SiNCs show very interesting electrical and optical properties. Their ability to trap charges could be exploited to create new generations of memory devices [17]. As for their optical properties, they could be employed in solar cells [18], and light amplification [3,19]. Optical and electrical properties are strongly dependent on the SiNCs structural characteristics: their size, their distribution, and the nature of their cluster/matrix interfaces. Consequently, a perfect control of the SiNCs growth during the elaboration and an accurate characterization of these parameters are crucial toward developing future potential applications and optimization of elaboration processes.

### Si nanoclusters elaboration

SiNCs are usually grown from a silicon-rich silicon oxide (SRSO). SRSO can be produced with many techniques such as ion implantation [20], CVD [21] or magnetron sputtering [22]. SRSO is then annealed to induce phase separation between silicon excess and silica. An efficient way to control the size of SiNCs is to produce SRSO thin layer between two SiO_2 _layers. These two silica layers prevent the Si excess from diffusing out the SRSO layer and restrict the diameter of produced SiNCs. Thus, a ML configuration is commonly adopted. In this study, SRSO/SiO_2 _MLs have been synthesized by reactive magnetron sputtering. A SiO_2 _pure target is sputtered on 1 cm^2 ^[100] Si substrates maintained at 500°C. Silica films are deposited under Ar plasma, while for the SRSO ones, a mixture of 50% H_2 _+ 50% Ar gas is used. In such conditions, SRSO layers contain approximately 50 at.% of silicon, i.e., Si excess is estimated to be 25 at.%. The thickness of each layer is tuned by the sputtering time. This deposition process is fully described in the reference [22]. MLs are subsequently annealed at 900°C for 1 h under N_2 _to favor the phase separation and the SiNCs growth. The thickness of each layer was accurately measured by high-resolution transmission electronic microscope (HRTEM) after the deposition process. It was estimated to be 4 nm for SiO_2 _layers, and 3.8 nm for SRSO layers. Specimens are then mounted on tip-shaped stainless steel needles for being analyzed with APT.

### SiNCs characterization

**Figure 3:**
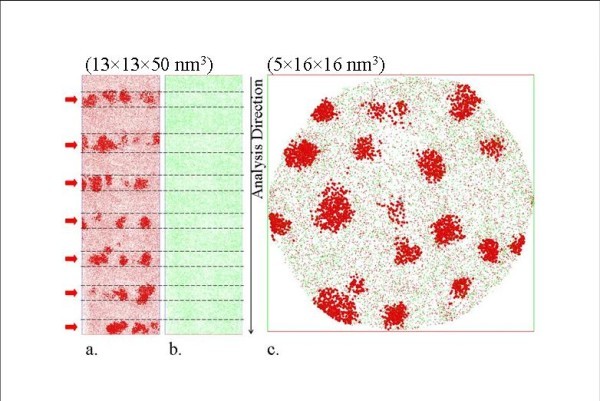
**Three-dimensional reconstruction of SRSO/SiO_2 _multilayer containing SiNCs**. a. Distribution of silicon and b. oxygen atoms in the analyzed volume. Each dot corresponds to one atom. Silicon atoms belonging to SiNCs are artificially magnified for clarity. Arrows indicate the location of SRSO layers. c. Cross-sectional view of a SRSO layer.

Conventional analysis methods, such as PL measurements or HRTEM, are usually performed to characterize SiNCs. However, these techniques suffer from serious drawbacks, such as the complete detection of all the crystalline and amorphous clusters. The use of APT technique allows us to overcome most of these drawbacks and characterize all the SiNCs (amorphous or crystalline). The local composition measured by counting silicon and oxygen atoms for the SiO_2 _layers gives a composition of 34.3 ± 0.3 at.% of Si which is in close agreement with the theoretical composition (33.3 at.% of Si). SRSO layers contain 51.0 ± 0.3 at.% of Si which is the expected value following the elaboration process (50 at.% of Si). The ability of APT to measure local composition at the atomic scale allows studying the phase separation within these SRSO layers. Figure 3a, b represents the 3 D reconstruction image of the SRSO/SiO_2 _MLs. Each dot represents Si (red) and O (green). The Si atomic map (Figure 3a) evidences that precipitation occurs in the SRSO where two phases can be distinguished: Si-clusters and SiO_2_-matrix. The local composition measurements allow us to detect SiNCs, and a cluster identification algorithm is used for highlighting all the atoms which are surrounded by at least 75 at.% of Si. Thus, all the clusters, crystalline or amorphous, are evidenced. In addition, the silicon concentration in the matrix is significantly higher than in pure silica with a value reaching 41.9 ± 0.3 at.%. Silicon excess is still present in the matrix evidencing an incomplete phase separation between Si and SiO_2 _after an annealing treatment at 900°C during 1 h. This result confirms the slow phase separation process at such temperature as previously reported in similar systems [23,24]. Finally, the SiNCs appear to be homogeneously distributed in the SRSO layers (Figure 3c).

**Figure 4:**
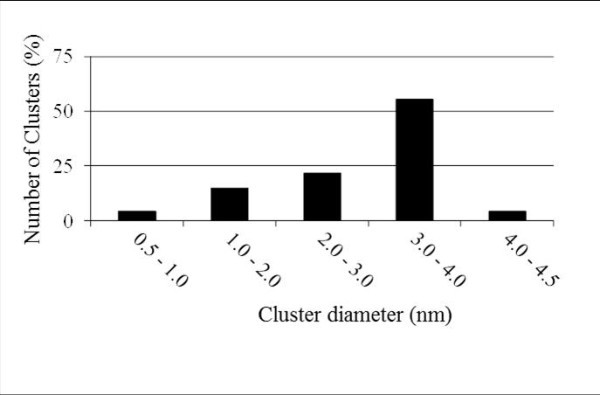
**Size distribution of SiNC measured in the SRSO layers of the analyzed volume**.

Size distribution of SiNCs can be accurately established with the detection of all the crystalline and amorphous silicon precipitates. Figure 4 shows the SiNCs size distribution. The mean diameter of SiNCs is 2.9 nm and sizes range from 0.5 to 4.5 nm. Most of SiNCs diameters lie in the range of 3.0-4.0 nm which corresponds to the thickness of the SRSO layer indicating that Si atoms in excess diffuse only in the SRSO layers. SiO_2 _layers act as diffusion barriers as predicted. The number density of particles is estimated to be 9.0 × 10^18 ^± 1.0 × 10^18 ^cm^-3^. This density is very close to the theoretical density number of particles which can be calculated if all the Si excess forms a precipitate of 3.8-nm diameter (layer thickness: 11.5 × 10^18 ^cm^-3^).

## Conclusions

In summary, the ATP has been used for the investigation of SiNWs and SRSO/SiO_2 _MLs containing SiNCs. An efficient sample preparation for APT is presented in both cases. The Au droplet on top of SiNW is reconstructed, and the core concentration of Au atoms is less than 5 ± 0.5 × 10^17 ^at./cm^3^. It is shown that B atoms can be uniformly distributed in the core of SiNW with a measured high concentration value of 1.4 ± 0.1 × 10^20 ^B/cm^3^. We have shown that APT also provides information on SiNCs which are inaccessible with the conventional analytic methods and particularly with HRTEM. The local composition of each layer and each phase as well as the structural properties of SiNCs can be investigated in a very accurate manner. It is shown that in the case of a 3.8-nm-thick SRSO layer with 25% of silicon in excess, a 1-h annealing treatment at 900°C induces the precipitation of SiNCs with a mean diameter of 2.9 nm. After this annealing treatment, the SRSO layers still contain 13% of silicon excess, evidencing an incomplete phase separation. These measurements show that APT is an efficient technique for the investigation of the phase separation in SiO_2_/SRSO MLs and the structural properties of SiNCs embedded in silica. Such a study is crucial to correlate electrical and optical properties with structural properties.

## Abbreviations

APT: atom probe tomography; CVD: chemical vapor deposition; FIB: focused ion beam; HRTEM: high-resolution transmission electronic microscope; PL: photoluminescence; PSD: position sensitive detector; SEM: scanning electron microscope; SiNWs: silicon nanowires; SiNCs: silicon nanoclusters; SRSO: silicon-rich silicon oxide.

## Competing interests

The authors declare that they have no competing interests.

## Authors' contributions

MR and WHC wrote the manuscript. MR and ET carried out the APT sample preparation by SEM-FIB, performed and interpreted the APT experiments concerning SiNCs. FG deposited the samples and performed HR-TEM experiments concerning SiNCs. WHC, RL and EC carried out the APT sample preparation by SEM-FIB, performed and interpreted the APT experiments concerning SiNWs. BG and DS carried out the elaboration of SiNWs. PP supervised the study and participated in the analysis of the results. All authors read and approved the manuscript.
